# Genomic selection in loblolly pine - from lab to field

**DOI:** 10.1186/1753-6561-5-S7-I8

**Published:** 2011-09-13

**Authors:** Fikret Isik, Ross Whetten, Jaime Zapata-Valenzuela, Funda Ogut, Steven McKeand

**Affiliations:** 1 Cooperative Tree Improvement Program, North Carolina State University, Raleigh, USA

## Background

Tree breeding is logistically complex and expensive, and breeders have long sought to use molecular markers to accelerate breeding. A candidate gene approach based on testing for association between the presence of DNA sequence variation in or near candidate genes, and phenotypic variation in a population has long been explored [1,2]. However, using candidate gene approach (QTLs) has not been successful in breeding [3,4]. QTL-trait associations detected in one genetic background are often not observed in other families, because of recombination of genes during the segregation and low levels of linkage disequilibrium in the population. A new technology called genomic selection (GS) is revolutionizing dairy cattle breeding. In GS, marker effects are first estimated in a large training population (>500) with both phenotypic and genotypic data. Subsequently, estimated marker effects are used to predict breeding values in validation populations for which marker genotypes but not phenotypes are available [4]. Several dairy cattle breeding companies now routinely use GS to select and market bulls. The success of GS in cattle breeding is largely based on bovine genome sequencing and discovery of thousands of SNP markers. GS application, if successful, will have a great impact on forest tree breeding because of their complex and logistically difficult breeding programs. Although, there have been several simulation studies examining the effective population size, linkage disequilibrium, and heritability on the predicted accuracy of GS in tree breeding [5], GS has not yet been demonstrated for forest trees using empirical markers data, mainly due to lack of sufficient dense markers.

## Methods

Biallelic SNP markers provided by the CTGN project (http://dendrome.ucdavis.edu/ctgn/) were used for genotyping. A population of 149 cloned full-sib offspring of loblolly pine (*Pinus taeda* L.) was phenotyped. Fitting 3406 informative SNP markers simultaneously, we estimated genome-wide breeding values and compared them with breeding values based on pedigree model. Variances explained by the marker additive and dominant effects were obtained.

## Results

The accuracy of the genomic estimated breeding values ranged from 0.30 to 0.83 for growth and wood quality traits. Lignin and cellulose content had great accuracy values from GS compared to growth traits. The accuracies were comparable with breeding values that were calculated based on the traditional pedigree model. If we take into account time needed to complete progeny testing, GS would be more efficient than classical progeny testing for some traits. The marker additive effects explained 18% and 23% for lignin and cellulose, respectively. Variances could not be determined for height and volume, because the Gibbs sampler failed to converge, even after five million iterations. We speculate that observed accuracies in this study trace familial linkage rather than historical LD with trait loci, because of small population size and relatively deep pedigrees. The markers are sampling the haplotypes and thus constructing the pedigrees rather than explaining phenotypic variance. Nevertheless, the results are promising, and we expect that with decreases in genotyping cost, GS has a potential to fundamentally change tree breeding in the near future.

## Challenges of GS applications in tree breeding

Despite promising results from some early work based on empirical data, there some challenges to overcome to routinely use GS in tree breeding. Conifers have genome size with a range between 18,000 and 40,000 Mbp [6]. Their populations have low levels of LD which decays rapidly. LD in loblolly pine decays to less than r^2^=0.25 within 2000 bp [7]. Low LD is due to genetic recombination over the evolutionary history of the species and causes inconsistency of QTL-marker association. Large genome size and historically low LD require large numbers of dense markers to explain a considerable amount of phenotypic variation in complex traits.

Another challenge is the lack of genetic maps in forest trees. With a few exceptions, the genomes of forest trees have not been sequenced, and thus precise locations of SNP markers are lacking, which hinders the use of haplotypes. Using haplotypes reduces the dimensions of the data and thus requires much smaller computing resources to analyze. More importantly, with haplotypes, larger variation between trees can be obtained using allelic combinations, although larger training populations are required to adequately sample the effects of all the haplotypes.

High marker genotyping cost is the major obstacle in applications of GS in forest trees. More cost efficient genotyping technologies, such as genotyping-by-sequencing and restriction digestion are being explored to reduce cost of markers. On the other hand, advances in computer power have made it possible to analyze large amount of complex data, but bioinformatics challenges still remain to analyze sequence data and SNP marker calling.

Further research is needed in development of training models and calibration of prediction model. The number of generations that statistical models can be used before losing accuracy remains to be determined in forest trees. Another question is the validity of models across different populations. In cattle breeding, lower accuracies of GS for dairy versus beef cattle remains a challenge. For some tree species, GxE interaction could be an issue to be addressed; observed marker-trait association observed in one population may not hold in another environment.

## Conclusions

We are currently working on construction of realized genomic relationship matrix based on SNP markers to use in predictions of breeding values. This method provides flexibility in terms of fitting common environmental effects in mixed models. We expect that decreases in marker genotyping costs will make GS in pine breeding feasible in the near future. Our group will work on pilot projects with forestry companies in the southern US, and plans are underway to revise breeding strategies to incorporate genomic selection.

## References

[B1] Tabor HK Risch NJ Myers RM Candidate-gene approaches for studying complex genetic traits: practical considerations Nat Rev Genet 2002 3 391 397 11988764 10.1038/nrg796

[B2] Hayes BJ Bowman PJ Chamberlain AJ Goddard ME Invited review: Genomic selection in dairy cattle: progress and challenges J Dairy Sci 2009 92 433 443 10.3168/jds.2008-1646 19164653

[B3] Goddard ME Hayes BJ Genomic selection J Anim Breed Genet 2007 124 323 330 10.1111/j.1439-0388.2007.00702.x 18076469

[B4] Meuwissen THE Hayes BJ Goddard ME Prediction of total genetic value using genome wide dense marker maps Genetics 2001 157 1819 1829 11290733 10.1093/genetics/157.4.1819 PMC1461589

[B5] Grattapaglia D Resende M Genomic selection in forest tree breeding Tree Genetics and Genomes 2010 7 2 241 255

[B6] Morse AM Peterson DG Islam-Faridi MN Smith KE Magbanua Z Evolution of genome size and complexity in Pinus PloS ONE 2009 4 2 e4332 doi:10.1371/journal.pone.0004332 10.1371/journal.pone.0004332 19194510 PMC2633040

[B7] Brown GR Gill GP Kuntz RJ Langley CH Neale DB Nucleotide diversity and linkage disequilibrium in loblolly pine PNAS 2004 101 15255 15260 10.1073/pnas.0404231101 15477602 PMC524047

